# Intra-household joint decision making on child feeding and associated social determinants in rural districts of South Ethiopia: a multi-site concurrent mixed method study

**DOI:** 10.3389/fpubh.2024.1381068

**Published:** 2024-12-12

**Authors:** Kassahun Fikadu, Abinet Takele, Biruk Tesfaye, Zeleke Hailemariam Abebo

**Affiliations:** ^1^Department of Midwifery, College of Medicine and Health Science, Arba Minch Universiy, Arba Minch, Ethiopia; ^2^School of Medicine, College of Medicine and Health Science, Arba Minch Universiy, Arba Minch, Ethiopia; ^3^Maternal and Child Health Department, Ethiopian Ministry of Health, Addis Ababa, Ethiopia; ^4^School of Public Health, College of Medicine and Health Science, Wachemo University, Hossana, Ethiopia

**Keywords:** barriers, joint intra-household decision making, socio-economic, South Ethiopia, factors

## Abstract

**Background:**

Improving joint intra-household decision-making by spouses is a promising solution to improve child-feeding practices. Therefore, this study aimed to assess the status and barriers of intra-household joint decision making on child feeding in rural districts of South Ethiopia from the perspectives of primary caregivers and key individuals.

**Methods:**

A mixed-method study was conducted from July 15 to September 15, 2023 in three randomly selected rural districts: Arba Minch Zuria, Mierab Abaya, and Chencha, in Southern Ethiopia. We employed a cross-sectional study design to collect quantitative data. A computer generated random number technique was used to include 25% of the total kebeles from each district. A total of 20 kebeles; 8 from 32 kebeles of chencha, 6 from 24 kebeles of Mierab Abaya, and 6 from 24 kebeles of Arba Minch Zuria were included. A total of 1,479 women with their children aged 6–23 months were recruited from family folder of the health extension program using a simple random sampling technique. A descriptive qualitative study design was used to collect data from 51 focus group discussants and 12 key informants. Trained health professionals collected the data using a structured and pre-tested interviewer administered questionnaire and semi-structured interviewer guide for quantitative and qualitative data, respectively. Univariate analysis was used to determine the frequency and percentages while Binary logistic regression analysis was employed to identify the associated factors. The odds ratio with a 95%CI was computed to assess strength of the association. The principles of saturation were adhered during the qualitative data collection. Thematic analysis was used to analyze the data in themes and subthemes, using ATLAS.ti version 23.

**Results:**

Overall, more than two-thirds (69.2%) of the intra-household decision-making regarding child feeding were found to be jointly decided by the spouses. Women with formal education (AOR = 1.84, 95% CI: 1.37–2.46), husband involvement in child feeding (AOR = 2.23, 95%CI: 1.70–2.92), having fewer than or equal to three children (AOR = 1.51, 95%CI: 1.11–2.04), women aged 25-34 years (AOR = 1.57, 95%CI: 1.19–2.07) and 35-49 years (AOR = 2.14, 95%CI: 1.38–3.33) were significantly associated with joint decision-making regarding child feeding practices. Moreover, large family sizes, large age gaps between husbands and wives, and gender inequalities were identified as barriers to women’s engagement in intra-household decision making based on qualitative analysis.

**Conclusion:**

In the rural districts of southern Ethiopia, joint intra-household decision making regarding child feeding was found to be satisfactory. Maternal education, husband’s involvement in child feeding, mother’s age, and number of children were independent variables that significantly influenced women’s engagement in joint intra-household decision making on child feeding. Interventions targeted at improving women’s engagement in decision-making should consider the following sociocultural barriers: extreme age differences between couples, large family sizes, and detrimental gender-oriented norms.

## 1 Introduction

Joint intra-household decision making is an essential driver of child health and nutrition outcomes, as one dimension of women’s empowerment determines how resources are allocated inside the household (1). Women’s involvement in family decision making is an essential predictor of better nutritional outcomes in newborns and young children (2). Women’s empowerment sometimes happens through navigating relational and societal dynamics through processes such as negotiation and manipulation during intra-household decision making (3). A systematic review and meta-analysis reported that, mothers involved in decision making were positively associated with recommended minimum dietary diversity feeding practices in Ethiopia. In Ethiopia mostly child feeding is the responsibility of mothers; therefore, involvement of mothers on their household matters can empower mothers to feed diversified diet for their child (4).

A variety of theoretical frameworks can be employed to analyze household decision-making, each offering unique insights into family dynamics. Notable models include the Collective Model, which acknowledges individual preferences and bargaining power that result in Pareto-efficient outcomes (5, 6), and the Unitary Model, which views decision-making as a unified process, overlooking the complexities present in households with multiple adults. The unitary model simplifies household decision-making as one entity with shared preferences, but critics say it overlooks complexities in multi-adult households (5, 7). Research in rural Ethiopia shows decisions in these households are not made by one person and lack Pareto efficiency. Spousal disagreement on women’s roles is common (5). The Non-cooperative game theory perspective (8): This Theory sees decision-making as a strategic interaction aimed at maximizing utility, while bargaining Models examine how household members negotiate resource distribution based on their bargaining power, which is shaped by income and social norms (5). Additionally, scholars can develop conceptual frameworks that integrate socio-economic status, gender roles, and cultural norms to better understand purchasing behaviors (9).

In Ethiopia, household decision-making dynamics are shaped by gender roles, socio-economic status, and cultural norms, with only about 24% of women holding significant decision-making authority (10). However, there is a noticeable shift toward more collaborative decision-making between spouses (11, 12). The Gender Roles and Social Norms Framework is especially relevant, emphasizing the significance of women’s autonomy in decision-making and its connection to better health outcomes for both women and children socio-economic status (13). The framework also can effectively captures the community and cultural complexities of decision-making processes within households (14), accounts socioeconomic factors such as education, wealth, and employment status, which significantly influence women’s decision-making power (9, 14, 15), and can inform policies and interventions aimed at enhancing women’s empowerment and improving health outcomes in the region (16).

Improving joint decision-making in intra-household decisions has been identified as a way of transforming the power-relations between men and women (17), thereby contributing to both women’s empowerment and improved development outcomes. This understanding has influenced development efforts aimed at improving rural livelihoods. Targeting couples’ decision-making, rather than women or men separately (18).Evidence shows that a higher level of decision-making power among rural women is linked with children’s feeding practices; improvement of reproductive, neonatal, and child health; and increased expenditures on household health and nutrition (19, 20). Moreover, it has been proven to be associated with increased nutritional diversity in homes (19, 21).

Global policy and development initiatives acknowledge the need to enhance joint decision-making authority among households (22). According to Gender Action Learning System (GALS), centering on the decision-making process of couples, as opposed to individual men or women, represent a fundamental element of gender equity, which has been executed in development initiatives across diverse countries (23).

The past decade has increased attention to measuring women’s empowerment and autonomy, motivated largely by the goal of identifying promising programs and policies for reducing gender inequalities. For the first time, the empowerment of women and girls is included in the Sustainable Development Goals as a stand-alone target (10, 24). In the social sciences, most approaches to defining and measuring empowerment are based on the concept of agency, defined by “ability to use those capabilities and opportunities to expand the choices they have and to control their own destiny,” and focus on women’s ability to participate in decision making over certain important matters.” (24).

However, empirical investigation of decision-making has revealed substantial heterogeneity across decision-making domains (25, 26). Decisions made either jointly or individually, are subject to varied dynamics depending on the context. According to two different contexts analysis, women in Bangladesh believe that they will achieve better productive outcomes if their partners have some input and decision-making authority over productive domains and thus associate autonomy more strongly with joint decision making (24). A similar conclusion was reached in a study examining decisions over contraceptive use, whereby women who were able to engage in “egalitarian” decision making (defined as involving discussion and agreement with their partners) were more likely to have positive contraceptive use out-comes as compared to women who made decisions independently (27).

This phenomenon can be observed in households that comprise a spouse or parental figures, such as a father, mother-in-law, or even offspring, where decisions are more likely to be in collaboration owing to the sharing of resources and responsibilities among household members (1, 28).The other factor in the decision-making process is the influence of prevailing social norms (28). Where social norms are disproportionately patriarchal and disparities in gender abounds, it can be anticipated that if the father does not participate in feeding, he might not pay enough allowances to the mother for her children’s food (29).

In rural Ethiopian households, it is common for three generations to coexist: 1. the older couples; 2. their sons, daughters-in-law, and unmarried daughters, and 3. grandchildren of married sons (30). Hence, it may be helpful to keep in mind that these household dynamics lead to an unequal distribution of resources and affect the health and nutritional status of mothers and children (10). It was demonstrated that gender inequality is both a cause and consequence of hunger and malnutrition in Ethiopia, and is associated with higher rates of both acute and chronic under nutrition. Furthermore, an equitable distribution of decision-making responsibilities is highly advantageous in interpersonal relationships between spouses. Therefore, as the nutritional benefits of increased income are determined by who controls the income and how it is distributed within the household, income alone is not enough to address under nutrition (10).

Previous studies in Ethiopia have focused on women decision making autonomy, without considering joint decision making specific to context to promote women empowerment. Although previous studies highlighted that variables such as: socio-demographic characteristics, child characteristics, maternal and child health characteristics, and household characteristics had correlation with women engagement in intra-household decision making (31–37), they lacked a comprehensive examination of how quantitative findings relate to sociocultural norms and beliefs in these contexts. Moreover, qualitative methods could provide profound insights into the lived experiences of individuals within households, revealing the complexities and nuances often overlooked in quantitative studies. Therefore, this study aimed to assess joint intra-household decision-making status and its socio-cultural factors in rural districts of southern Ethiopia.

## 2 Methods

### 2.1 Study setting and design

This study was conducted in three randomly selected rural districts of southern region of Ethiopia, Gamo zone. Gamo zone is located 450 kilometers distance from capital of the country, Addis Ababa with both highland and lowland topography. The three districts: Mirab Abaya, Arba Minch Zuria, and Chencha, were randomly selected to represent the eight districts in the zone. According to the 2023 data of the respective districts, Mirab Abaya district had 24 rural kebeles with 15,584 households, Arba Minch Zuria district had 24 kebeles with 25,047 total households, and Chencha district had 32 rural kebeles with 12,618 households. These districts were located in the Gamo zone of southern Ethiopia and had a total populations of 295, 882, according to the 2007 national census data projection of Ethiopia. A community-based, concurrent, mixed study design was conducted using both quantitative and qualitative approaches. Quantitative and qualitative data were collected using a community-based cross-sectional study design and a descriptive qualitative study design, respectively. Both quantitative and qualitative data were collected from July 15 to September 15, 2023.

### 2.2 Sampling methods

Study participants were selected by simple random sampling technique using health extension workers’ family folder and the participants were interviewed face to face. FGD discussants and KII participants for qualitative data were recruited based on the rich information they had about the context of decision making in the area.

### 2.3 Study method

Quantitative data were collected using a closed-ended interviewer-administered questionnaire prepared from different studies on household decision-making (38–41). This method was used because of the literacy level of study participants to understand the questionnaire, as the study was conducted at rural households most study participants might not understand self-administered questionnaire. The qualitative data were collected by prepared interviewer-administered guide.

### 2.4 Study population and sampling technique

The source population for this study was married women with children aged 6–23 months living in the rural districts of southern Ethiopia. Married women aged 15–49 years who had 6–23 months old children and lived for at least 6 months in the study area with their husbands at the time of the study were enrolled, while single mothers living with their children were excluded. This population was targeted because of the aim of the study was to determine the joint decision making status of the households about infants and young children complementary feeding. Study participants were selected using a multistage sampling technique because of the nature of the study requires sampling at three steps: at district, kebele level and at household level. Three rural districts were randomly selected from eight districts in the zone. Then, 25 % of the kebeles from each district: six kebeles from 24 rural kebeles located in Mirab Abaya district, six kebeles from 24 kebeles located in Arba Minch Zuria district, and eight kebeles from 32 kebeles located in Chencha district were randomly selected. Then, considering all the eligible households at each district, the total calculated sample (1502) was proportionally allocated to each district followed by proportional allocation again to each kebele. The number of households with children aged 6–23 months at each kebele was used for proportional allocation. Consequently, the allocated numbers of samples were drawn by a simple random sampling technique using computer-generated random numbers from the family folder at each kebele level. Finally, community leaders were used as a guide to reach out to households. Purposive sampling was used to select participants and information saturation was used to limit the sample size. The purpose was to find out information rich sources about facilitators and barriers of joint intra-house hold decision making.

### 2.5 Determination of sampling size

The total sample size was calculated using a single-population proportion formula from an online open source (Open Epi version 3) for quantitative data. We calculated the required sample size using the following assumptions: population size (*N*) = 1,000,000, hypothesized % frequency of outcome factor in the population (*p*) = 20% (10), confidence limits as % of 100(absolute +/− %)(*d*) = 3, 95% confidence interval, and design effect (for cluster surveys-*DEFF*) = 2 because of the level we went through to reach out the study units. Hence, relatively the largest sample size was 1,502. As 98.4% of the participants responded for the study, 1,479 participants gave information for this study. However, since non-response rate was considered during sample size determination and design effect was considered for the multistage sampling, the sample included in this study was adequate. For qualitative data, six focus group discussions (FGD), each consisting of eight to nine discussants and four in-depth interviews from each district, a total of 12 KII with religious leaders, community elders, women representatives in the community, and health extension workers were conducted. The sample size for qualitative data was decided based on the information saturation principle.

### 2.6 Variable measurements and study instruments

The dependent variable of the study was joint household decision-making (1 = joint intra-household decision-making among the spouses, 0 = Intra-household decision making was done without involvement of either spouse).

**Joint intra household decision-making** was measured by asking the participants for the following three questions: determining their own healthcare, making major household purchases, and visiting their family or relatives. Each question had six responses: (1) women had a final decision after discussing with their husband, (2) women and husband decided jointly, (3) husband’s final decision, (4) women and other family members, (5) another family member alone, and (6) others. We dichotomized response 1 and 2 into one category as ‘Joint decision’. In this group, the classification was made if the decision is made either after discussion where both have sufficient understanding or the less dominant one does not feel disempowered as long as the partner had sufficient understanding and intention (42) and recoded it into a score of ‘1.’ The second group was made for the rest of the responses as ‘No joint decision’ and it was given a score of ‘0’ (33). The mean score was calculated to dichotomize the three variables into one composite variable. Thus, a score above the calculated mean of 2.55 were considered having joint decision-making, otherwise not.

The **independent variables** in this study were socio-demographic characteristics (age categories of the mother, religion of the mother, educational status of the mother, educational status of the husband, occupational status of mother, occupational status of husband), child characteristics (age, sex, episode of known new-born sickness, complementary feeding initiation time, and breastfeeding status), maternal and child health characteristics (parity, ANC follow-up, place of delivery, postnatal care attended, number of children, husband involvement in infant feeding), and household characteristics (drinking water source, knowledge of infant feeding practices, mothers’ attitudes toward infant feeding practice).

**Knowledge about infants and young child feeding (IYCF)**: This was measured using five items measuring child feeding-related knowledge. Based on the summation score, a score of less than 60% considered as low knowledge, a score of 60–75% average knowledge, and a score greater than 75% was high knowledge (41).

**Women’s attitude** toward child feeding in this study was measured as: There were seven questions overall. The scoring order is as follows (5 = strongly disagree, 4 = disagree, 3 = neutral, 2 = agree, and 1 = strongly agree). The total score is calculated by computing the values for each item. Finally, the score ≥ of the mean value was considered ‘favorable attitude,’ while the corresponding was taken as ‘unfavorable’ attitude’ (38).

**Improved source of water**: Water from piped, boreholes or tube wells, protected dug wells, and protected springs sources, while an unimproved source of water was obtained from unprotected dug wells or springs and surface water (e.g., lakes, rivers, streams, ponds, canals, and irrigation ditches) (43).

**Household food security**: Was assessed using the Food Insecurity Experience Scale (FIES), developed by the Hungary initiative of the United Nations Food and Agricultural Organization to enhance simplicity and acceptability demands (44, 45). It has eight items: (1) worried about not having enough food; (2) Could not eat healthy and nutritious food); (3) Only a few kinds of foods; (4) Had to skip a meal; (5) Ate less than you thought you should; (6) Household ran out of food; (7) Were hungry but did not eat; and (8) Went without eating for an entire day. All cases responding ‘No’ were coded as 0 and those with a repose ‘Yes’ was labeled in to ‘1.’ Likewise, Food Secure was declared if the response to all items were ‘No’ or ‘Yes’ for the first questions. Thus, [(Q1 = 0 or Q1 = 1) and Q2 = 0 and Q3 = 0 and Q4 = 0 and Q5 = 0 and Q6 = 0 and Q7 = 0 and Q8 = 0 and Q9 = 0] are considered food-secure. If at least one of the following criteria was fulfilled: (1) if [(Q2 = 1 or Q3 = 1 or Q4 = 1) and Q5 = 0 and Q6 = 0 and Q7 = 0 and Q8 = 0 and Q9 = 0]; (2) if [(Q5 = 1 or Q6 = 1) and Q7 = 0 and Q8 = 0 and Q9 = 0]; or (3) if [Q7 = 1 or Q8 = 1] (39). Qualitative data were collected through focus group discussions (FGD) and an in-depth interview guide developed from different studies (46, 47).

### 2.7 Data quality assurance

Twenty B.Sc. holder data collectors and three M.Sc. holder supervisors collected the data after 5 days of training on how to use the application and the meaning of each variable. The data collectors were clinical professionals with experience in application-based data collection. The data collectors covered 6–7 households per day. The closed ended questionnaire developed by reviewing different related literatures. The questionnaire had five sections such as socio-demographic, knowledge, attitude, food security and, maternal and child health related characteristics. The Kobo-collect application-based questionnaire was pre-tested in 5% of mothers in the study area, which was not included in the actual study, and necessary corrections were made such as the decision-making questionnaire was reduced from eight to three for the convenience of the participants’ understanding. Moreover, we checked Cronbach alpha to test the reliability of the questionnaire. Accordingly, the overall Cronbach alpha value was 7.3, 7.8 and 8.5 for knowledge, attitude and intra-household decision making questionnaires, respectively. All questionnaires were checked daily for accuracy and cleaned before analysis. Participants were asked to have a private room or space to reduce social desirability bias. Five data collectors collected qualitative data, and the data collectors were M.Sc. with experience in qualitative data collection and fluent speakers of the local language. Key informant interviews were held at the offices, each lasting 30–45 min, and audio-recorded. The FGD lasted for 60–80 min, and the discussions were audio-recorded. Participants engaged in informal conversations in the form of unstructured, spontaneous discussions to obtain the opportunity to ask pertinent questions on different occasions. This could minimize the possibility of participants purposefully altering their responses or holding back information on sensitive issues.

The focus group discussion continued till saturation was first perceived. The mood of the focus group participants was the indication followed to data saturation. Data saturation was obtained when participants had no additional ideas to contribute during discussion. Furthermore, saturation was also noticed during coding of the focus group transcripts. We confirmed data saturation by reading and re- reading transcripts and developing initial codes. During thematic analysis, coding stopped when no new codes emerged and was considered as indication of data saturation (48). Data saturation was maintained during transcript’s coding by assigning different coders, the two authors (KF, ZHA), and whenever there was a difference in codes, the authors decided the coding saturation by discussion.

### 2.8 Data management and analysis

Quantitative data were exported from the KoboCollect server to SPSS version 22 and analyzed. Univariate analysis was performed for frequency and used to determine the joint decision making status of the households along with other independent variables. All regression assumptions were checked before running logistic regression. Multicollinearity was checked using the variance inflation factor (VIF), and model fitness was checked using the Hosmer-Lemeshow goodness of fit test. The full model has a significant prediction performance (X^2^ = 91.89; df = 9; *p* < 0.001), and the model also has a good fit because the Hosmer and Lemeshow Test could not reject the hypothesis of model appropriateness, as Chi-square was 4.84 and *p* = 0.68. The model correctly classified 70.5% of cases overall. All variables with a *p*-value less than 0.25 were selected and fitted to the final logistic regression model to identify variables associated with joint decision making status. In multivariable regressions, *p*-values less than 0.05, at a 95% confidence interval, were considered a cutoff to declare variables as predictor variables using stepwise backward regression. Transcriptions of the qualitative data were obtained from audio recordings, and field notes were used to characterize the participants. The transcripts were first read several times to obtain an overall picture, and then translated. Transcripts were analyzed using ATLAS Ti9 software and to familiarize with the data, we read and reread the transcripts. After familiarization with the interviews, we conducted initial open coding independently (KF and ZH) and reviewed it by two independent experts (AT and BT). The development of themes were determined by the content of the interview guides combined with the inductive development of codes as they emerged from the data. After the themes were identified from the codes, connections across themes were identified through ordering and re-ordering using the ATLAS Ti9 software. Using an iterative process, revisions and corrections were made to the codebook to reflect emerging themes throughout the analysis. The identified themes were discussed and structured. The results were interpreted to answer the following research question: socio-cultural factors contributing to joint decision making of spouses.

## 3 Results

### 3.1 Socio-demographic characteristics of study participants

A total of 1,479 mother–child paired respondents participated in this study, with a response rate of 98.4%. The mean age of the mothers was 27.31 (SD = 5.78) years. Of study participants, More than half (52.7%) of the mothers were 25–34 years old. More than one third (37.6%) of the mothers and one third (32.5%) of the husbands attended primary education. More than three quarters (77.4%) of the mothers and more than one half (55.8%) of husbands were housewives and farmers, respectively. Four in five of the households (83.3%) had improved sources of water for drinking and food preparation (Table 1).

**Table 1:** Socio-demographic characteristics of the study participants at rural districts of south Ethiopia, 2023.

Variables	Frequency (*n* = 1,479)	Percent (%)
Age categories of the mother		
15 to 24	475	32.1
25 to 34	780	52.7
35 to 49	224	15.1
Religion of the mother		
Protestant	900	60.9
Orthodox	536	36.2
Others^a^	43	2.9
Educational status of the mother		
Unable to read and write	324	21.9
Able to read and write	167	11.3
Primary school	556	37.6
Secondary school	318	21.5
College and above	114	7.7
Educational status of the husband		
Unable to read and write	205	13.9
Able to read and write	196	13.3
Primary school	481	32.5
Secondary school	391	26.4
College and above	206	13.9
Occupational status of mother		
Housewife	1,145	77.4
Merchant	123	8.3
Farmer	89	6.0
Government employee	63	4.3
Private employee	30	2.0
Others^b^	29	2.0
Occupational status of husband		
Farmer	825	55.8
Merchant	203	13.7
Daily laborer	183	12.4
Government Employee	123	8.3
Private employee	145	9.8
Water sources of the household		
Improved	1,232	83.3
Unimproved	247	16.7

a Catholic, Muslim.

b Daily laborer, weaver.

### 3.2 Characteristics of participants in the qualitative interviews

A total of 63 individuals participated in the study for both the six FGDs and 12 KIIs. The mean age of the study participants was 34.9 (SD ±6.45) years (range, 22–50 years). More than half (58.7%) of the participants were between 30 and 40 years of, and half (50.8%) of the participants were female. Majority (55.6%) of the participants had secondary or higher educational status. Nearly half (47.6%) of the participants were government employees by occupation (Table 2).

**Table 2:** Socio-demographic characteristics of qualitative study participants, South Ethiopia, 2023.

Characteristics	Frequency	Percentage
Age in years		
20–30	18	28.6
31–40	37	58.7
41–50	8	12.7
Sex		
Female	32	50.8
Male	31	49.2
Educational status		
Had no formal education	4	6.3
Primary	24	38.1
Secondary plus	35	55.6
Occupational status		
Farmer	17	27.0
Government employer	30	47.6
Merchant	7	11.1
Private employer	9	14.3

### 3.3 Knowledge, attitude, food security, and breast feeding related characteristics

As for complementary feeding, greater number of mothers started complementary feeding 1,447 (97.8%), and most started the complementary feeding after 6 month of the child’s age 1,204 (83.2%). Related to household food security, out of the total 1,479 households 1,275 (86.2%) were found to be food insecure. Most of the mothers, 956 (64.6%), with children aged less than 2 years, had high knowledge of complementary feeding. Of the total of 1,479 mothers, 807 (54.6%) had favorable attitudes toward complementary feeding (Table 3).

**Table 3:** Knowledge, attitude, food security, and breast feeding related characteristics of the study participants, south Ethiopia, 2023.

Variables (*N* = 1,479)	Frequency (n)	Percentage (%)
Ever heard of child feeding practice		
Yes	1,240	83.8
No	239	16.2
Ceased breast feeding		
No	1,244	84.1
Yes	235	15.9
Started complementary feeding		
Yes	1,447	97.8
No	32	2.2
Complementary feeding starting time		
After six months	1,204	83.2
Up to six months	243	16.8
Food security		
Food secure	204	13.8
Food insecure	1,275	86.2
Knowledge on complementary feeding		
Good Knowledge	956	64.6
Average knowledge	363	24.5
Poor knowledge	160	10.8
Attitude on complementary feeding		
Favorable Attitude	807	54.6
Unfavorable attitude	672	45.4

### 3.4 Maternal and child health related characteristics

More than half (54.4%) of the women were multipara, and 1,417 (95.8%) of the women had antenatal care follow-up during their recent pregnancy. Approximately 902 (61%) attended postnatal care. Approximately 70 percent of households had fewer than four children (Table 4).

**Table 4:** Maternal and child health related characteristics of participants living in rural distrits in south Ethiopia, 2023.

Variables		Frequency(n)	Percent (%)
Parity	Primiparous	352	23.8
	Multiparous	869	58.8
	Grand multiparous	258	17.4
ANC follow up	Yes	1,417	95.8
	No	62	4.2
Place of delivery	Home	206	13.9
	Health center	954	64.5
	Public hospital	311	21
	Private clinic	8	0.5
Postnatal care attended	Yes	902	61
	No	577	39
Number of children	One to Three	1,043	70.5
	Four and Above	436	29.5
Husband involvement in infant feeding	Yes	1,175	79.4
	No	304	20.6
Sex of children	Male	804	54.4
	Female	675	45.6
Age of children (months)	6–11	460	31.1
	12–17	479	32.4
	18–23	540	36.5
Episode of the child sickness	Yes	1,043	70.5
	No	436	29.5

### 3.5 Intra-household decision making related to child feeding practices

**Figure 1:**
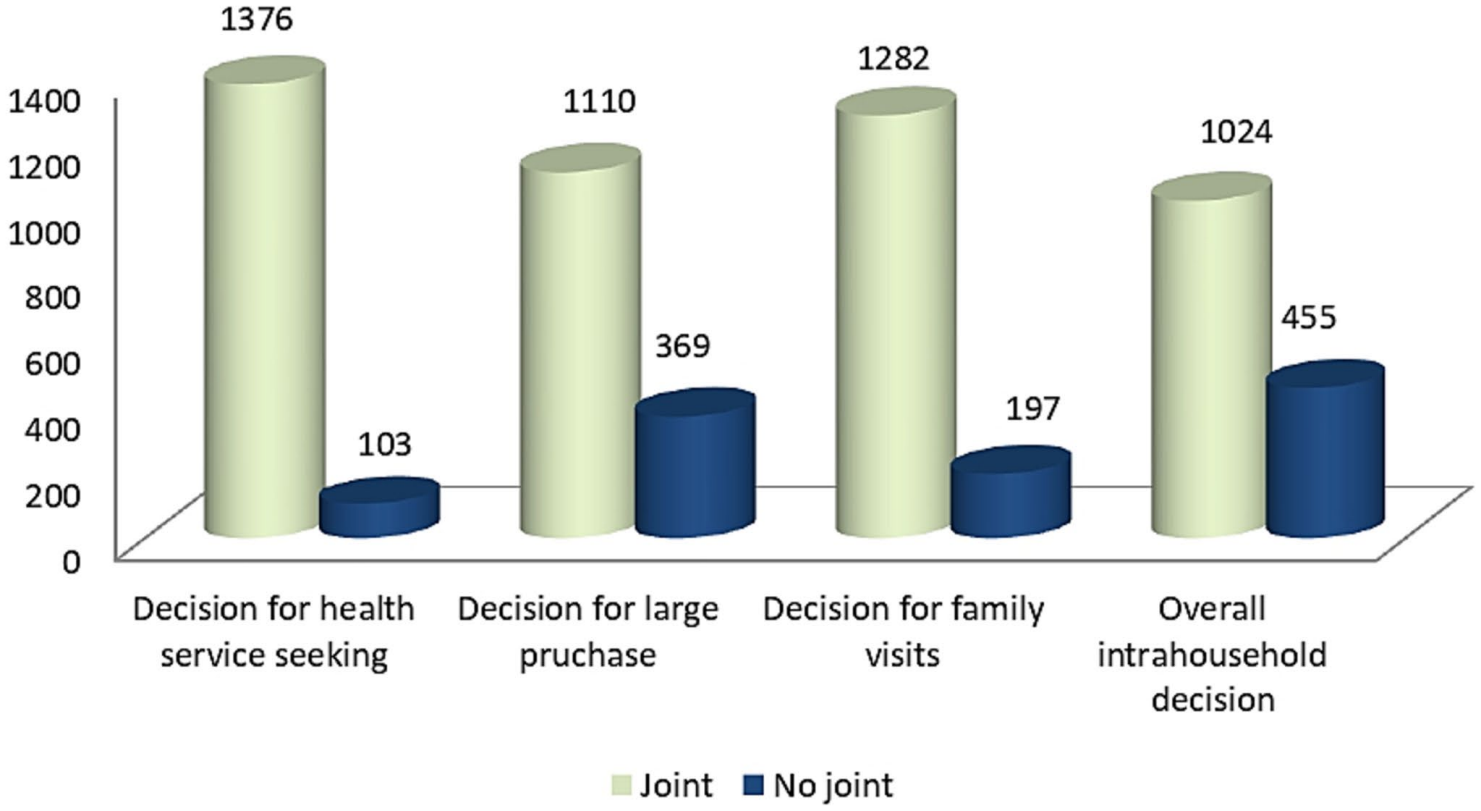
Status of intra-household decision making in rural districts of South Ethiopia, 2023.

Most of the study participants, 1,024 (69.2%; 95CI: 60, 77.8%) were involved in joint household decision-making, while 455 (30.8%) of the mothers reported no joint participation. Regarding health services seeking decisions, 1,374 (93%) participated jointly with their husbands. Three-quarters of the participants jointly decided on large household purchases. In the majority of households, 1,282 (86.7%) decisions regarding family visits were jointly made (Figure 1).

### 3.6 Factors/barriers associated with women’s decision making participation on child feeding practices

Logistic regression was performed to identify the variables associated with household decision-making. Variables such as age, mother’s educational status, husband’s educational status, mother’s occupation, husband’s occupation, access to television or radio, household food insecurity, number of children per household, husband’s involvement in child feeding, sex of the child, episodes of child sickness, mother’s cessation of breastfeeding, complementary feeding started, mothers’ attitudes toward child feeding, mothers’ knowledge of child feeding, and parity affected joint intra-household decision-making.

The full model has a significant prediction performance (X^2^ = 91.89; df = 9; *p* < 0.001), and the model also has a good fit because the Hosmer and Lemeshow Test could not reject the hypothesis of model appropriateness, as Chi-square was 4.84 and *p* = 0.68. The model correctly classified 70.5% of cases overall. Therefore, maternal education, husband’s involvement in child feeding, mother’s age, and number of children significantly determined women’s participation in household decisions (Table 5).

**Table 5:** Factors associated with joint intra-household decision making in rural districts of South Ethiopia, 2023.

Variables	Household decision making		Crude odds ratio with 95% confidence interval	Adjusted odds ratio with 95% confidence interval	*p*-value
	Joint	Solo			
	Number (n)	Number (n)			
Mother education					
Formal education	837	318	1.93(1.49–2.49)***	**1.84(1.37–2.46)**	0.00
No education®	187	137	1	1	
Husband involved in child feeding					
Yes	864	311	2.5(1.92–3.24)***	**2.23(1.70–2.92)**	0.00
No®	160	144	1	1	
Husband occupation					
Farmer	542	283	0.62(0.37–1.04)	0.64(0.37–1.11)	0.11
Merchant	155	48	1.04(0.57–1.89)	0.99(0.54–1.84)	0.99
Civil servant	149	37	1.30(0.70–2.41)	1.01(0.53–1.91)	0.98
Day Laborer	116	67	0.56(0.31–1.00)	0.66(0.36–1.22)	0.19
Others*®	62	20	1	1	
Mother’s age					
25–34	555	225	1.27(0.99–1.62)	**1.57(1.19–2.07)**	**0.002**
35–49	155	69	1.15(0.82–1.62)	**2.14(1.38–3.33)**	**0.001**
15–24®	314	161	1	1	
Household food insecurity					
Secured	555	252	0.95(0.76–1.19)	1.36(0.96–1.94)	0.08
Not secured®	469	203	1	**1**	
Number of children					
One to Three	747	296	1.45(1.14–1.84)**	**1.51(1.11–2.04)**	**0.01**
Four and Above	277	159	1	**1**	

NB: ®reference group, *Weaver, ** < 0.01, *** < 0.00.

Three main themes were generated from the FGD and KII data that explain the barriers to women’s participation in decision-making regarding child feeding practices in rural districts in southern Ethiopia. The themes were economic, demographic, and socio-cultural.

Mothers who attended formal education were twice as likely to participate in joint household decision-making as compared to those who did not attend formal education (AOR = 1.84, 95% CI: 1.37, 2.46).

Key informants and participants in the FDG discussion reported that educated women are able to make joint decisions about what they think is important to their children, whereas illiterate women give their children what recipes are easily found at home.

*“There is a difference between educated and uneducated women. Since educated women are able to plan for the needs of their newborns, including the duration of breastfeeding and the appropriate diet for the child and on the other hand, uneducated women tend to take the fact of being alive as a blessing, educated women convince their husbands about child nutrition and get engaged in resource allocation for household food consumption, particularly for children.* [43 years old male FG discussant].

*“Educated and uneducated women were not the same. Thus, educated mothers could prepare different recipes. However, an uneducated person consumes and eats what is available in the house. If adults eat corn, they can feed their babies only with corn. The level of education of mothers affects their dietary decisions.”* [A 30 years old female KI].

*“She [the educated woman] has a positive influence on children’s health and nutrition decisions. An educated mother has a positive influence on her children. Because she knows how to take care of them, wash them, dress them, clean them, and treat them well.”* [A 36 years old female KI].

The women whose husbands involved in child feeding was two times more likely to practice joint intra-household decision making than those did not (AOR = 2.23, 95%CI: 1.70, 2.92).

Findings from the qualitative method showed that when men are engaged in caring for their children and sharing their family responsibilities, it provides a space for women to make joint decisions with their husbands.

*“Men think washing and feeding the children is her [wife’s] task; therefore, he does not get engaged. When giving money to buy food items, men limit the amount, then she (the wife) says this is not enough, and he says, ‘How not enough?’ A few days later, if she asks him again, he says, the last day you bought much, where did it go? …. This hampers her decision-making capacity and freedom*.” [A 40 years old male FG discussant].

*“Some husbands are not willing to give already prepared ready feeding for their children rather they wait until she [the wife] comes back from where she goes. It is my duty to keep children when the mother has some task or if she goes to social issues.”* [A 35 years old male FG discussant].

*“…Yes, it [husband involvement in child feeding] affects joint decision making on children’s feeding. Since our area is in a rural setting where there is a lack of awareness about many things, for example, in rural areas, men do not cook lunch......... If the children at home are hungry, but he sits and watches, do not attempt to work on feeding them. Therefore, he may not understand what to purchase for children”* [43-year-old female, KII participant].

Women above the age of 25 to 34 years were 1.5 times more likely to participate in intra-household decision making about child feeding as compared to aged less than 25 years of age women (AOR = 1.57, 95%CI: 1.19, 2.07). Older mothers aged 35 to 49 are 2.14 times (AOR = 2.14, 95%CI: 1.38, 3.33) more likely to participate in decision making with their spouse compared to younger women aged less than 25 years.

The majority of the respondents from the qualitative study reported that when the age of the women was smaller, their husbands loved to order and even insulted the younger women they married. Since husbands are older adults, wives submit themselves but with conflicts of interest against the decisions made by husbands.

*“…A small age is not necessarily related to irrelevant thinking. She [women at a younger age] may have a good idea, but because of age differences, her husband would like to order, insult, and prescribe everything in the house. Communication, dealing, dialogue, and discussions were highly affected. …., therefore good communication habits are important.*” [A 39 years old male FG discussant].

*“Women who are younger may say things like, “I am a child; I do not want to suffer for my child if there is communication difficulty.” When there is little to no age gap, women put up with their husband’s behavior, even when it makes him angry. When the husband and wife argue, the younger wife takes her frustrations out on the kids and neglects to take care of them….”* [A 33 years old female FG discussant].

The findings also showed that women with fewer children were 1.5 times more likely to participate in household decision making in relation to child feeding practices than those with four or more children (AOR = 1.51, 95%CI: 1.11–2.04).

Most focus group participants explained that when family size increases, there is an imbalance between a household’s consumption needs and available resources. There is no cash to feed children. If there are several children living at home, the situation worsens.

*“If the number of children is too much, the need is very high; therefore, there is much missing between need and supply. It is not easy to feed many children. For example, our work is ‘shema’ (waving), and when a social issue happens we stop working for three or more days. There will be no money for child’s food. This condition complicates what women should decide for the child.”* [A 32 years old female FG discussant].

*“Many children in one household is really difficult to manage, the older children took the youngster’s food. Mother should have followed them, but mother takes care of the younger. But most women go to market prepare food for the children and put in the house, the older children eat all the food, the younger stay without food. In this case women are not decision maker with the limited resources they have*” [A 29 years old female FG discussant].

## 4 Discussion

In numerous sub-Saharan African nations, such as Ethiopia, men hold primary control over household decision-making, resulting in women being placed in a subordinate position across all decision-making aspects (49, 50). This includes their ability to make decisions regarding their child’s feeding practices, which is crucial as they are the primary caregivers for infants and young children (51). Research has shown that when women have greater authority in decision-making within the household, including the purchase of significant household assets, daily necessities, freedom of movement, and their own healthcare, it has a positive impact on child-feeding practices (52). However, women’s involvement in decision making related to major household purchases is predominantly determined by their husbands (49). In Ethiopia, the majority of women reside in rural areas, where sociocultural barriers may hinder their decision-making regarding infant and young child-feeding practices (53, 54). Therefore, this study aimed to determine the status of joint decision-making and the associated sociocultural barriers in rural districts of the southern Ethiopian region.

The joint decision-making on infant feeding practices in the present study accounted for 69.2%. Aligning with a similar study conducted in Mizan Aman, South West Ethiopia, where the percentage was 67.2% (37), a study conducted in Dawro Zone 64.2% (55) a secondary analysis based EDHS 70.55% (34), northwest Ethiopia (75.1%) (32), pooled prevalence 70% (56), Ghana (75%) (33)and a study conducted in India (68%) (57). Furthermore, this finding was higher than those in Ghana (52.8%) (2), Nigeria (38.9%) (31), Nepal (47.1%) (58), Senegal (6.26%) (43), and Pakistan (28%) (36). The variation in the socio-demographic profiles of the settings could explain the observed discrepancies. For instance, a study conducted in a low-income country revealed that, as individuals age, their awareness of household matters increases, leading to greater involvement in decision-making processes (56). Additionally, differences in the timing of studies, the level of attention given to the issue, and the existence of policies and strategies aimed at promoting women’s autonomy in household decision making contribute to the observed variation. A study carried out in Senegal provides additional insights into the impact of sociocultural norms on women’s decision making. This highlights that the majority of decisions (80.3%) (59) are made by husbands, indicating their dominant role (60) influenced by specific cultural norms (35, 61). Consequently, this limits women’s participation in decision-making processes, particularly concerning their freedom of movement, access to healthcare services, and making choices regarding significant purchases or essential needs for their children.

This study also discovered a significant statistical association between joint decision making and educational status. This finding is in agreement with several studies conducted in Ethiopia (34, 41), Senegal (59), Zambia (35), Nepal (58), and Ghana (33). This implies that educated women are more likely to possess the necessary personality, decision-making ability (62), mobility, and directly contribute to the socioeconomic development of households (49), and help build self-confidence (62) to negotiate on matters they perceive they should take part in, which also provides them with equal decision-making roles and the possibility of being employed. On the other hand, the current study also outlined education as one of the barriers hindering collaborative decision-making, which indicates that the attainment of education empowers women to have increased involvement in household decisions (63). Overall, educated women can be aware of their right to freedom of movement and are capable of exercising it as far as child-feeding is concerned. Empowering women with education is a crosscutting issue for key stakeholders such as the Ministry of Education and other program implementers.

Husband/partner involvement in child feeding was found to be an independent factor affecting women’s decision-making in the household. This was supported by a multinational study conducted in a sub-Saharan African setting. Partner involvement positively empowered women to actively participate in household decision-making (64). In Nepal, partner or husband participation is strongly associated with joint decision making (65). This suggests that increased women’s participation in household matters works in synergy with their husbands’ involvement, mainly related to child-feeding practices. It also provides the insight that couples that used to cooperate in household decisions are likely to view child feeding as a shared practice. In fact, the enhanced availability of food for children in the household can be achieved through husbands’ engagement rather than by nominating children feeding a role assigned to women (66). Thus, women with active participation in household matters are more likely to attain their preferences, and this can assist implementation strategies, as husbands’ participation in child feeding is viewed as an acceptable norm and also benefits couples without underestimating women’s decision-making. In contrast to its positive implications, disengagement of the husband is one of the barriers to this study. In the current study, decision-making power was hindered by the lack of collaborative or shared roles in the household. This was supported by a study in Gambia, which revealed that the patriarchal nature of a household can influence women’s right to make decisions regarding resource allocation, education, and childcare (47).

This study also shows that women’s decision-making in household matters, particularly related to child-feeding practices, was positively associated with participants’ age, indicating that advanced women’s age influences women’s decision-making in rural households. Several studies conducted in Albania (67), Senegal (59), rural Ethiopia (68), Zambia (35) and Nepal (58) support this finding. This indicates that the tradition of viewing older women has an important or influential position in society, which implies a change in the role of women as their age advances (69). However, compared to men, the view of society toward women of the same age generally lacks recognition of providing equal value or position, regardless of their age (70). In support of this, the current study also revealed that age differences between couples could be a barrier to the active participation of women in household decision-making. This implies that women no longer tend to feel shy or ashamed as they age; rather, they feel confident and find it difficult to express their thoughts freely and participate in household decision-making. In line with the current study, qualitative studies conducted in India (71), Columbia (72), and Ethiopia (73) also showed that the influence of age variation on the active participation of women in household decision-making is a viable factor shared by these countries.

Another finding of this study is that women with fewer children are more likely to participate in household decision making. In agreement with the quantitative findings, the FGD discussant in the qualitative section of this study also outlined that having a larger number of children in the family is a barrier to household decision-making. This could be because of the scarcity of resources to be shared, including time spent with children, which makes the mother worried more about choosing what her baby needs. This observation shows that women living in regions with high fertility are likely to suffer from low participation in household decision-making (74). However, this explanation can also imply that for women living in patriarchal households, having children, especially sons, can help improve their participation in household matters (75). In contrast, a qualitative study from South Asia showed that women’s participation in household decision-making can be enhanced in the presence of daughters, which is consistent with a theoretical model in which mothers have greater relative preferences for spending on their daughters than fathers, and thus seek more autonomy to direct resources to their daughters (76). Although the literature has argued the importance of the sex of the child, we do not know whether a large number of daughters or sons will leave women with autonomy unaffected (77). Nevertheless, women who exercise active participation either by themselves or under circumstances are less likely to need many children as a prop for their status. This is support by the “theory of allocation of time,” proving the idea that there has been a shift from investing in the quantity of offspring to investing in their quality, as proposed by Gary Becker and his colleagues (78). This concept works for the decision-making process of parents who face resource constraints, such as time and money, which is common in developing nations, such as Ethiopia. Educated women are more likely to opt for fewer children because they understand the opportunity cost of childbearing, which falls mainly on them (79). The costs of having more children, in terms of productivity and time opportunity costs, increase with each additional child.

### 4.1 Strengths and limitations

Data were collected using both quantitative and qualitative approaches and employed the perspectives of husbands, wives, and other information sources. This study employed joint decision-making questions from large-scale studies, which could improve the validity of the measurement. We employed a large sample size, which could enhance the generalizability of our findings to a similar context; however, the identified context specific socio-cultural factors in this study may not be generalizable to other settings. Moreover, the findings of this study in general might not represent the urban contexts as we collected the data from rural districts. The study participants were selected from health extension program’s family folder this might have affected the actual random selection. Due to the self-reported response, we also acknowledge the social desirability bias; to reduce this bias, a short questionnaire was used over a large number of questions. As this study was observational in nature, the identified associations did not guarantee causality between independent and dependent variables, hence, further research in the area should focus on experimental and longitudinal studies. Suggest future research using longitudinal or experimental designs to explore causal relationships. Qualitative data were collected by focusing on socioeconomic determinants, which limited the exploration of more information from qualitative data sources.

## 5 Conclusion/recommendation

This study found that the majority of participants reported joint decision-making. It is imperative to note that women’s joint participation in household decisions is highly affected by their age, educational status, number of children, and husbands’ involvement in child-feeding. Programs aimed at promoting shared decision-making—especially those that include educational initiatives and engage men in childcare—should be given due attention in policy goals. This could involve the education sector in favor of improving women’s literacy or family guidance in providing family planning services to limit the number of children. Furthermore, programs targeting women and children should also consider male involvement.

## Data Availability

The original contributions presented in the study are included in the article/supplementary material, further inquiries can be directed to the corresponding author.
